# Associations of Urinary Cadmium with Age and Urinary Proteins: Further Evidence of Physiological Variations Unrelated to Metal Accumulation and Toxicity

**DOI:** 10.1289/ehp.1306607

**Published:** 2013-06-07

**Authors:** Agnes Chaumont, Catherine Voisin, Gladys Deumer, Vincent Haufroid, Isabella Annesi-Maesano, Harry Roels, Lutgarde Thijs, Jan Staessen, Alfred Bernard

**Affiliations:** 1 Laboratory of Toxicology and Applied Pharmacology, Catholic University of Louvain, Brussels, Belgium; 2 Université Pierre et Marie Curie (Paris 6), Medical School Saint-Antoine, Paris, France; 3 INSERM (Institut national de la sante et de la recherche medicale) U707 EPAR (Epidémiologie des maladies allergiques et respiratoires), Paris, France; 4 Division of Hypertension and Cardiovascular Rehabilitation, University of Leuven, Leuven, Belgium; 5 Department of Epidemiology, Maastricht University, Maastricht, the Netherlands

## Abstract

Background: The current risk assessment for environmental cadmium (Cd) largely relies on the assumption that urinary Cd (U-Cd) is a reliable biomarker of the Cd body burden. Recent studies have questioned the validity of this assumption.

Objectives: We studied the lifetime trend of U-Cd as a function of diuresis, gender, smoking status, and protein tubular reabsorption. We also analyzed the associations between U-Cd and urinary proteins.

Methods: Cd, retinol-binding protein, and albumin were measured in the urine of six cohorts of the general population of Belgium, with a mean age ranging from 5.7 to 88.1 years (*n* = 1,567). Variations of U-Cd with age were modeled using natural cubic splines.

Results: In both genders, U-Cd decreased to a minimum (~ 0.20 μg/L) at the end of adolescence, then increased until 60–70 years of age (~ 0.60 μg/L in never-smokers) before leveling off or decreasing. When U-Cd was expressed in micrograms per gram of creatinine, these variations were amplified (minimum, 0.15 µg/g creatinine; maximum, 0.70 µg/g creatinine) and much higher U-Cd values were observed in women. We observed no difference in U-Cd levels between never-smokers and former smokers, and the difference with current smokers did not increase over time. Lifetime curves of U-Cd were higher with increasing urinary retinol-binding protein or albumin, a consequence of the coexcretion of Cd with proteins.

Conclusions: At low Cd exposure levels, U-Cd and age are associated through nonlinear and nonmonotonic relationships that appear to be driven mainly by recent Cd intake and physiological variations in the excretion of creatinine and proteins.

Citation: Chaumont A, Voisin C, Deumer G, Haufroid V, Annesi-Maesano I, Roels H, Thijs L, Staessen J, Bernard A. 2013. Associations of urinary cadmium with age and urinary proteins: further evidence of physiological variations unrelated to metal accumulation and toxicity. Environ Health Perspect 121:1047–1053; http://dx.doi.org/10.1289/ehp.1306607

## Introduction

Cadmium (Cd), a by-product of zinc production, is one of the most cumulative and toxic metals to which humans can be exposed. The two major sources of Cd exposure for the general population are diet and tobacco smoking. Once absorbed by ingestion or inhalation, Cd efficiently accumulates in the organism, in particular in the kidneys, where it is retained with a biological half-life exceeding 20 years. As a consequence, the Cd body burden, which is negligible at birth, rises continuously during life until 60–70 years of age, then levels off or even decreases. (Nordberg et al. 2007). The current consensus is that in chronic Cd intoxication the kidney, the main site of storage, is also the critical target organ (i.e., the first organ to be affected). The earliest adverse effect of Cd is an impairment of the tubular function, resulting in an increased urinary excretion of low-molecular-weight (LMW) proteins such as retinol-binding protein. This tubular dysfunction develops in a dose-dependent manner, with the risk of LMW proteinuria appearing only when the Cd concentration in kidney cortex (K-Cd) reaches a threshold of 150–200 μg/g wet weight (Nordberg et al. 2007).

From a toxicological point of view, Cd presents a rather unique property in that the amount of Cd stored in the kidneys can be estimated noninvasively by measuring the Cd concentration in urine (U-Cd). Studies in industrial workers have shown that, before renal dysfunction occurs, there is a significant correlation between U-Cd and K-Cd (Roels et al. 1981, 1983). This finding was rapidly extended to the general population, an extrapolation based on the assumption that the relationship between U-Cd and K-Cd holds, regardless of the route, level, and duration of exposure (Buchet et al. 1990; Friberg 1984). To our knowledge, only two studies have checked the validity of this assumption. Nilsson et al. (2000) found no significant association between U-Cd and K-Cd as determined by an X-ray fluorescence technique in a group of Swedish farmers. Orlowski et al. (1998) found a significant association, but this was in an autopsy study using urine samples contaminated with Cd from bladder autolysis.

Based on the premise that U-Cd reflects Cd body burden, several studies have been conducted over the past few years among the general population. These studies have reported a wide range of health effects associated with low-level U-Cd, including renal dysfunction, bone demineralization and fractures, reproductive impairment, neurodevelopmental deficits, cancers, and even mortality [for review, see Nordberg et al. (2007) and Järup and Åkesson (2009)]. Recent studies, however, have reported observations that challenge the long-held view that U-Cd is a reliable biomarker of the Cd body burden at low exposure levels. Basically, these studies show that U-Cd is strongly influenced by a series of factors unlikely to be related to Cd toxicity or accumulation. For instance, Weaver et al. (2011) demonstrated a positive correlation between U-Cd and the glomerular filtration rate (GFR), which most likely reflects faster elimination of Cd with more efficient renal function. Another factor contributing to U-Cd variations is the residual influence of diuresis that persists after correction for urinary creatinine (U-Creat) or for specific gravity (Chaumont et al. 2011, 2012; Haddam et al. 2011). These studies also provided evidence of a coexcretion of Cd and proteins in urine as a result of the normal physiological variations in the tubular reabsorption of LMW proteins, including Cd-metallothionein (Cd-MT) (Akerstrom et al. 2012; Chaumont et al. 2012; Haddam et al. 2011). To further complicate the picture, tobacco smoking, long regarded as a mere additional source of Cd, may be able to cause changes in renal function, distorting the relationships between U-Cd and LMW proteinuria (Chaumont et al. 2011).

The objective of the present study was to reassess the significance of U-Cd as a biomarker of Cd body burden or cumulative exposure to the metal. We compared the lifetime trend of U-Cd as a function of gender, smoking status, diuresis, and urinary protein excretion. We also analyzed the associations between U-Cd and urinary proteins in different age groups, comparing them with the relationship described in an early Cadmibel study (Buchet et al. 1990).

## Materials and Methods

*Study populations*. For the present study, we used sets of data from six separate epidemiological studies conducted on the general population of Belgium. Three of the studies were performed in schools with the objective of evaluating the impact of environmental stressors on child or adolescent health. The first study was performed in 2006–2007 and included 847 adolescents from three secondary schools (mean age, 15.4 years) (Bernard et al. 2008; Chaumont et al. 2012); the second in 2007–2008 included 430 children in the third year of kindergarten (third kindergarten) in 30 schools (mean age, 5.7 years) (Voisin et al. 2010); and the third in 2009–2010 included 400 first-grade children in the same schools as for the 2007–2008 study (mean age, 7.6 years) (Voisin et al. 2013). The three other studies evaluated adults (age range, 18–98 years) living in the same areas. The first adult study, the Cadmibel study, was carried out in 1985–1989 in 1,220 participants and examined the health impact of environmental pollution by Cd (Buchet et al. 1990). The second adult study, conducted in 2001 with 272 participants, aimed to evaluate dioxin and heavy metal exposure in the vicinity of waste treatment facilities and iron and steel plants (Fierens et al. 2003). The third study in adults was performed in 2011 in 34 nursing home residents (Bentayeb M, Norback D, Bednarek M, Bernard A, Cai G, Cerrai S, Eleftheriou K, Gratziou C, Lavaud F, Sestini P, Sarno G, Sigsgaard T, Viegi G, Wieslander G, Zielisnki J, Annesi-Maesano I, unpublished data).

For the present study, we excluded adult participants suffering from diabetes or from chronic disease likely to affect renal function (*n* = 276), as well as those who had been occupationally exposed to heavy metals including Cd (*n* = 167). To minimize confounding by diuresis, we also excluded adult participants with U-Creat < 0.3 and > 3 g/L (*n* = 127). For children in third kindergarten and for adolescents, we randomly selected 200 subjects (both genders). For children in the first primary school, only 95 participants (both genders) were available who had not been enrolled when they were in third kindergarten. Two children were excluded because their concentrations of urinary retinol-binding protein (U-RBP) were outliers in the graph of residuals (102 and 667 µg/L). We also excluded six nonsmoking adults because their U-Cd were > 3 times the SD from the mean calculated for this group. The level of statistical significance was set at *p* < 0.05 (two-sided). Thus, the total population (*n* = 1,567) included all age groups between 4.8 and 98.0 years.

*Study protocol*. The protocols for all six studies were approved by the Ethics Committee of the Faculty of Medicine of Catholic University of Louvain. Adults provided written informed consent, and children and adolescents were examined only with the written consent of their parents. Study participants or parents, in the case of children and adolescents, filled out a self-administered questionnaire about age, gender, and factors likely to affect renal function and exposure to Cd. For all of the studies, body weight and height were measured and urine samples were collected. Participants in the Cadmibel study provided a 24-hr urine sample, but urine samples from the other studies were untimed. For children and adolescents, untimed urine samples were collected during school hours (between 0900 hours and 1600 hours)

U-Cd analysis. In all of the studies except Cadmibel (Buchet et al. 1990), Cd was measured in urine samples by inductively coupled argon plasma mass spectrometry (ICP-MS) with an Agilent 7500 instrument (Agilent Technologies. Santa Clara, CA, USA) (Chaumont et al. 2011). To ensure the comparability of data for the present study, we reanalyzed the urine samples of adolescents (*n* = 200) that had been previously analyzed by the University of Lund in the framework of the Phime project (Chaumont et al. 2012). Briefly, urine specimens (500 µL) were diluted quantitatively [1:9 (vol/vol)] with a 1% nitric acid/0.5% hydrochloric acid solution containing scandium, germanium, rhodium, and iridium as internal standards. The detection and quantification limits were 0.02 and 0.05 µg/L, respectively. In the Cadmibel study, U-Cd was determined by electrothermal atomic absorption spectrometry (Perkin-Elmer Zeeman-3030 or Zeeman-5100) using stabilized-temperature-platform-furnace techniques coupled with a Zeeman-effect background correction system (Lauwerys et al. 1990). (For the analytical performances of these methods, see Supplemental Material).

U-Creat analysis. For all studies except Cadmibel, U-Creat was determined by a modified Jaffé reaction using a Beckman Synchron LX 20 analyzer (Beckman Coulter GmbH, Krefeld, Germany) (Hare 1950). The concentrations of U-RBP and urinary albumin (U-Alb) were determined by latex immunoassay (Bernard and Lauwerys 1983). In the Cadmibel study, U-Creat was determined on a Cobas Bio centrifugal analyzer (Roche Diagnostics GmBH, Mannheim, Germany).

*Statistical analyses*. Data were analyzed using R software, version 2.14.2 (http://cran.r-project.org/bin/windows/base/old/2.14.2/). All biological parameters are reported as median and interquartile range (IQR) and were log-transformed to approximate normal distribution. We used Student’s *t*-test to examine gender differences with regard to U-Cd and other urinary parameters (U-Creat, U-RBP, U-Alb). The urinary excretion of biomarkers was compared across age groups and according to smoking status by one-way analysis of variance (ANOVA) followed by the Tukey-Kramer post hoc test. Variations of U-Cd and other urinary proteins with age were modeled using the natural cubic spline function. Knots were placed at fixed quantiles of the predictor’s marginal distribution as suggested by Harrell (2001). Selection of the number and location of the knots was based on minimizing Akaike’s information criteria. We used the function “ns” in R with the “splines” package to model the natural cubic spline. Urinary biomarkers are expressed per liter of urine and per gram of creatinine. The models were run by stratifying the population according to gender, smoking status, and renal handling of proteins as reflected by tertiles of U-RBP or U-Alb. The analysis for renal handling of proteins was performed by adjusting U-Cd, U-RBP, and U-Alb for the residual influence of diuresis on the basis of the simple linear regression coefficient between U-Creat and the biomarkers.

Associations of U-Cd with biomarkers of renal function were assessed by multiple linear regression. We applied stepwise forward selection to select covariables with significance levels of 0.25 for a variable to enter and 0.10 to stay in the model. The tested independent variables were body mass index (BMI), age, and gender. We performed these analyses by testing two different models to adjust for the residual effect of diuresis. In the first model, proteins and U-Cd were expressed per gram of creatinine; in the second model, the urinary concentrations of these biomarkers were expressed per liter. In both models, U-Creat was considered as a separate independent variable to adjust for the residual influence of diuresis, as suggested by Barr et al. (2005). The underlying statistical assumptions about the homoscedasticity and normality of the errors were verified visually with regression residuals. Independence of the residuals was assessed by the Durbin-Watson test.

## Results

Characteristics of the studied populations and the concentrations of U-Cd and other urinary biomarkers are summarized in Table 1 for children and adolescents. Tables 2 and 3 show data for adults and the elderly, separated according to smoking status and gender, respectively. The mean age of the studied populations ranged from 5.7 to 88.1 years, with an overall range extending from 4.8 to 98 years. When considering all groups except smokers, the median U-Cd levels ranged from 0.24 µg/L in children to 0.62 µg/L in the elderly, and from 0.16 µg/g creatinine in adolescents to 0.60 µg/g creatinine in the elderly. In adults, median U-Cd was higher in never-smoker women (0.46 µg/L; 0.57 µg/g creatinine) than in never-smoker men (0.36 µg/L; 0.27 µg/g creatinine) and higher in current smokers (0.64 µg/L; 0.67 µg/g creatinine) than in either former smokers (0.52 µg/L; 0.48 µg/g creatinine) or never-smokers (0.42 µg/L; 0.43 µg/g creatinine). We observed no differences in U-Cd between males and females of groups other than never-smokers, nor between former and never-smoker adults. In all age groups except the elderly, U-Cd expressed in micrograms per gram of creatinine was negatively correlated to U-Creat (children, *n* = 296, *r*^2^ = 0.171, *p* < 0.001; adolescents, *n* = 200, *r*^2^ = 0.073, *p* < 0.001; adults, *n* = 1,048, *r*^2^ = 0.108, *p* < 0.001; elderly, *n* = 23, *r*^2^ = 0.140, *p* = 0.08), indicating a residual influence of diuresis due to an overadjustment on the basis of U-Creat. The number of cigarettes smoked per day and the number of pack-years (available only for 358 current smokers; medians of 20 and 18, respectively) were positively correlated with U-Cd (*n* = 358, *r*^2^ = 0.033, *p* < 0.001 and *r*^2^ = 0.30, *p* < 0.001, respectively) and U-Alb (*n* = 330, *r*^2^ = 0.025, *p* = 0.004 and *r*^2^ = 0.033, *p* = 0.002, respectively) but not with U-RBP (*n* = 330, *r*^2^ = 0.0003, *p* = 0.92 and *r*^2^ = 0.003, *p* = 0.35) (urinary parameters are expressed per gram of creatinine).

**Table 1:** Characteristics and biological parameters of children and adolescents.

		Children				Adolescents	
		2007–2008		2010		2006	
		Boys	Girls	Boys	Girls	Boys	Girls
*n*		107	92	49	48	101	99
Age (years)		5.6 (4.8–6.8)	5.7 (4.9–6.3)	7.6 (6.0–8.9)	7.7 (6.7–8.8)	15.6 (14.0–17.8)	15.6 (14.2–17.3)
BMI (kg/m^2^)		20.7±3.0	20.4±3.5	17.4±2.2	17.2±2.8	20.7±3.2	20.6±2.6
U-Creat (g/L)		0.74 (0.58–0.93)	0.66 (0.51–0.90)	0.95 (0.83–1.27)	0.92 (0.63–1.11)	1.65 (1.16–1.96)	1.65 (1.30–2.0)
U-Cd	μg/L	0.24 (0.17–0.30)	0.23 (0.18–0.32)	0.29 (0.24–0.37)	0.23 (0.18–0.37)	0.24 (0.19–0.32)	0.27 (0.20–0.34)
	μg/g creatinine	0.32 (0.25–0.45)	0.36 (0.28–0.47)	0.31 (0.25–0.36)	0.32 (0.25–0.36)	0.16 (0.13–0.20)	0.16 (0.13–0.22)
U-RBP	μg/L	97 (70.0–157)	110 (78.0–140)	86.0 (63.0–119)	80.0 (42.0–123)	158 (100–269)	173 (107–265)
	μg/g creatinine	143 (103–206)	163 (131–207)	88.0 (65.5–114)	89.6 (60.3–127)	113 (74.4–152)	113 (80.9–150)
U-Alb	mg/L	1.80 (1.00–3.88)	2.70 (1.30–5.55)	3.50 (2.40–6.25)	5.85 (2.80–9.40)	5.72 (2.28–17.9)	9.68 (4.73–42.9)
	m/g creatinine	2.62 (1.45–4.71)	4.09 (2.27–7.48)	3.52 (2.28–5.96)	6.77 (3.02–11.3)	3.80 (1.83–11.0)	6.21 (3.42–26.3)
Values are mean (minimum–maximum) for age, mean±SD for BMI, and median (IQR) for urinary biomarkers.							

**Table 2:** Characteristics and biological parameters of adult population according to smoking status.

		Adults, general population						Elderly, nursing homes		
		1985–1989			2001–2002			2011		
		Never-smokers	Former smokers	Current smokers	Never-smokers	Former smokers	Current smokers	Never-smokers	Former smokers	Current smokers
*n*		331	155	333	146	52	31	13	6	4
Men [*n* (%)]		88 (26.6)	90 (58.1)	158 (47.4)	59 (40.4)	38 (73.1)	18 (58.1)	1 (7.7)	4 (66.7)	1 (25.0)
Age (years)		49.8 (19–88)	50.3 (20–85)	43.6 (18–80)	51.4 (25–71)	51.3 (33–80)	50.4 (33–69)	88.0 (79–98)	76.7 (70–88)	76.8 (61–83)
BMI (kg/m^2^)		25.1±4.4	25.9±4.4	24.1±4.2	26.3±4.9	27.2±3.5	25.9±4.3	22.4±2.2	23.3±6.3	21.3±1.0
U-Creat (g/L)		0.84 (0.59–1.23)	0.92 (0.69–1.22)	0.94 (0.62–1.45)	1.34 (0.96–1.70)	1.32 (1.05–1.90)	1.37 (1.01–2.07)	0.94 (0.62–1.67)	1.08 (0.77–1.24)	1.13 (0.59–1.74)
U-Cd	μg/L	0.40 (0.25–0.67)	0.49 (0.32–0.72)	0.62 (0.38–1.04)	0.56 (0.35–0.93)	0.68 (0.42–0.98)	1.12 (0.67–1.82)	0.62 (0.50–1.03)	0.82 (0.34–1.09)	0.91 (0.45–2.15)
	μg/g creatinine	0.49 (0.27–0.85)	0.52 (0.32–0.82)	0.67 (0.38–1.15)	0.43 (0.28–0.75)	0.45 (0.33–0.70)	0.68 (0.51–1.19)	0.60 (0.51–0.87)	0.56 (0.41–1.52)	0.95 (0.48–2.50)
U-RBP	μg/L	76.0 (48.0–130)	79.0 (50.0–130)	90.0 (55.8–155)	ND	ND	ND	108 (72.3–182)	107 (61.0–276)	85.5 (45.5–223)
	μg/g creatinine	91.0 (60.9–139)	81.7 (59.1–116)	97.1 (67.2–140)	ND	ND	ND	96.2 (71.3–235)	100 (76.9–134)	71.7 (43.1–167)
U-Alb	mg/L	8.12 (4.68–14.8)	6.38 (4.13–12.4)	7.52 (4.21–13.9)	ND	ND	ND	13.0 (5.75–42.3)	21.0 (13.0–170)	43.5 (4.50–143)
	mg/g creatinine	10.3 (4.06–24.2)	7.20 (3.75–18.1)	9.14 (3.28–19.6)	ND	ND	ND	19.7 (6.58–52.9)	23.8 (12.3–85.1)	33.1 (6.44–156)
ND, not determined. Values are mean (minimum–maximum) for age, mean±SD for BMI, and median (IQR) for urinary biomarkers.										

**Table 3:** Characteristics and biological parameters of adult population according to gender.

		Adults, general population				Elderly, nursing homes	
		1985–1989		2001–2002		2011	
		Men	Women	Men	Women	Men	Women
*n*		336	483	115	114	6	17
Age (years)		46.9 (19–88)	47.7 (18–88)	51.3 (30–80)	51 (25–73)	76.3 (61–88)	85.5 (71–98)
BMI (kg/m^2^)		25.2±3.5	24.6±4.9	26.3±3.6	26.4±5.4	24.2±5.9	22.3±1.9
U-Creat (g/L)		1.18 (0.81–1.58)	0.75 (0.55–1.10)	1.38 (1.05–1.90)	1.24 (0.93–1.63)	1.45 (1.11–2.01)	0.79 (0.62–1.48)
U-Cd	μg/L	0.52 (0.32–0.88)	0.47 (0.29–0.79)	0.52 (0.32–0.94)	0.72 (0.46–1.26)	0.96 (0.81–1.09)	0.62 (0.47–1.07)
	μg/g creatinine	0.50 (0.28–0.84)	0.62 (0.35–1.05)	0.38 (0.25–0.60)	0.65 (0.40–0.93)	0.66 (0.41–0.66)	0.60 (0.45–1.31)
U-RBP	μg/L	108 (61.0–160)	74.0 (46.0–120)	ND	ND	200 (95.0–387)	79.0 (65.5–142)
	μg/g creatinine	91.6 (61.7–133)	91.1 (63.3–139)	ND	ND	127 (47.2–233)	84.0 (71.3–185)
U-Alb	mg/L	5.20 (2.97–10.1)	9.95 (5.96–16.7)	ND	ND	26.5 (5.0–270)	13.0 (5.75–52.8)
	mg/g creatinine	4.91 (2.0–11.4)	13.4 (5.69–27.6)	ND	ND	18.7 (7.77–85.0)	19.7 (8.19–53.7)
ND, not determined. Values are mean (minimum–maximum) for age, mean±SD for BMI, and median (IQR) for urinary biomarkers.							

Even though all analyses were performed in the same laboratory, we ascertained the comparability of the Cadmibel data from 1985–1989 (Buchet et al. 1990) with values observed by Fierens et al. (2003) in the same areas > 15 years later by analyzing the relationships linking U-Cd to age. These relationships had virtually identical slopes and intercepts, as shown in Supplemental Material, Figure S1. The mean U-Cd concentrations adjusted for the age of 50 years did not differ between the two studies (0.48 for the Cadmibel population vs. 0.44 µg/g creatinine for the population recruited in 2001; *p* > 0.05).

**Figure 1:**
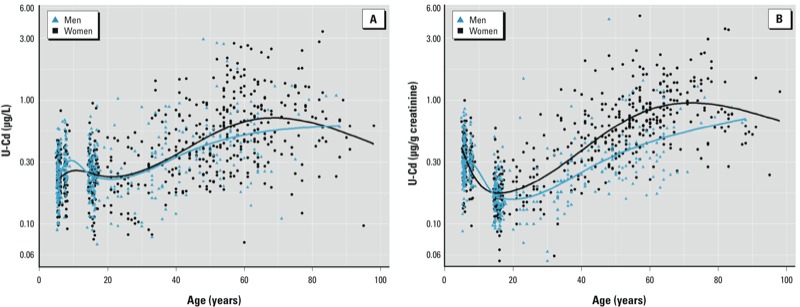
Associations of U-Cd in µg/L (*A*) or µg/g creatinine (*B*) with age according to gender in healthy and non­smoking male (*n* = 405) and female (*n* = 581) participants. Data were fitted using natural cubic splines with four knots placed at the 20th, 40th, 60th, and 80th percentiles. Differences between males and females were statistically significant (*p* < 0.05) at ≥ 60 years of age in (*A*), and at ≥ 20 years of age in (*B*).

Lifetime variations of U-Cd and proteins were derived from linear regression that included age as a natural cubic spline function and by combining all data except those from smokers. These models were run separately for men and women and for urinary parameters expressed per liter or per gram of creatinine. As shown in Figure 1, both models show that the relationship between U-Cd and age is nonlinear and nonmonotonic over a lifetime. U-Cd, expressed as micrograms per liter (Figure 1A), increased with age in both genders from 25 years of age (0.23 µg/L) to 60–70 years of age (0.64 µg/L), when U-Cd decreased in women and leveled off in men. During that period, U-Cd increased by a factor of about three in both genders. Before adulthood, there was little variation in U-Cd except for a small peak in children around 8 years of age. When U-Cd was expressed per gram of creatinine (Figure 1B), patterns of change with age were similar, but we observed more variation before adulthood and between men and women. In particular, we observed a 2-fold decrease of U-Cd in adolescents compared with children; from approximately 18 years of age, U-Cd became systematically lower in men than in women. The increase of U-Cd during adulthood was also amplified, with an average 4-fold increase of U-Cd between the ages of 20 and 60–70 years (0.16–0.72 µg/g creatinine).

The fitted curves of U-Creat and protein changes over a lifetime and according to gender are shown in Supplemental Material, Figure S2. Clearly in both males and females, U-Creat increases in childhood, reaches a maximum value at adolescence, and then steadily decreases with age—but much more abruptly in women, which creates a systematic gender difference over the rest of life. This gender-related variation is the opposite of that observed with U-Cd per gram of creatinine. Like for U-Creat, the excretion of U-RBP and U-Alb showed a sharp transient increase during adolescence, followed by a progressive increase with age. Although these two phases exist for U-RBP expressed as per liter and per gram creatinine, they seem to be preceded by a sharp decline in U-RBP excretion that occurs during early childhood.

**Figure 2:**
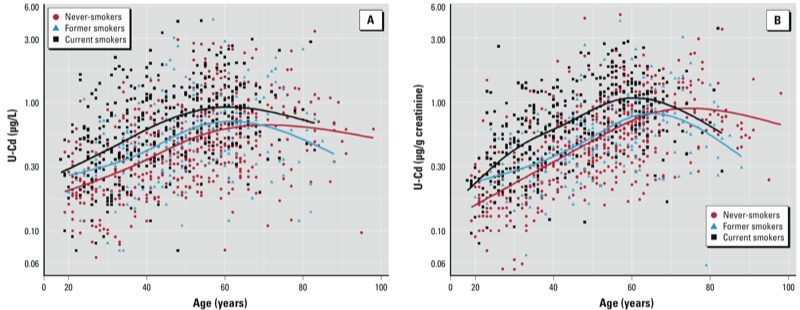
Associations of U-Cd in µg/L (*A*) or µg/g creatinine (*B*) with age according to smoking status. Data were fitted using natural cubic splines with three knots placed at the 25th, 50th and 75th percentiles. In adults 18–40 years of age, U-Cd of current and former smokers was significantly higher than that in never-smokers; however, after 40 years of age, the difference was statistically significant only between current and never-smokers. Never-smokers, *n* = 490; former smokers, *n* = 213; current smokers, *n* = 368.

To gain further insight into the relationships between U-Cd and the cumulative exposure to Cd, we compared the changes in U-Cd with age according to smoking status (Figure 2). U-Cd levels were systematically higher in current smokers than in never-smokers up to 70 years of age, but this difference remained constant, contrary to the expectation for a biomarker to reflect an increase in Cd body burden with chronic smoking. Indeed, at the 20, 40, and 60 years of age, mean U-Cd concentrations expressed per liter (or per gram of creatinine) of current smokers exceeded that of never-smokers by 45% (50%), 70% (76%), and 47% (49%), respectively. The pattern of U-Cd with age in former smokers did not show the expected difference with never-smokers, with the two groups reaching practically the same mean U-Cd level at 60 years of age. If anything, former smokers showed a lower mean U-Cd concentration after the age of 60.

**Figure 3:**
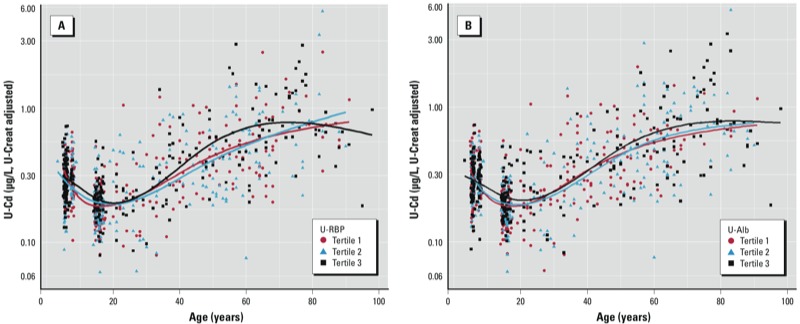
Association between U-Cd and age by tertiles of U-RBP (*A*) and of U‑Alb (*B*) in the nonsmoking population (*n* = 840). Data were fitted using natural cubic splines with three knots placed at 25th, 50th and 75th percentiles. U-Cd values were significantly different between tertiles for U-RBP and U‑Alb from adolescence.

We completed our analyses by evaluating the influence of renal handling of proteins on the lifetime patterns of U-Cd. First, we applied multiple linear regression analyses to assess the associations between U-Cd and U-RBP or U-Alb, expressed per liter or per gram of creatinine, in the different age groups. We observed no association between U-Cd and U-Alb in any of the models tested. In contrast, U-RBP was consistently associated with U-Cd and BMI among children, adolescents, and adults regardless of smoking status (Table 4). Paradoxically, associations between U-Cd and U-RBP were strongest in children and especially in adolescents (i.e., the age groups with the lowest median U-Cd levels). Second, we then compared the lifetime patterns of U-Cd in the whole population stratified by tertiles of U-RBP. We performed the same analysis with U-Alb because recent studies provided evidence of a coexcretion between Cd and albumin (Akerstrom et al. 2012; Chaumont et al. 2012). Variations in Cd intake were minimized by excluding former and current smokers. We also minimized the variations due to diuresis by adjusting both U-Cd and urinary proteins for the residual associations with U-Creat observed in each age group. Figure 3 shows that in both adults and adolescents there was a shift of U-Cd to higher values with increasing U-RBP and U-Alb. At 60 years of age, U-Cd reached a value that was approximately 30% higher in the highest tertile of U-RBP compared with the intermediate or lowest tertile (Figure 3A). U-Cd levels were similarly increased by the higher U-Alb excretion, with about a 20% difference between the highest and the lowest tertiles of U-Alb from puberty to 60 years of age (Figure 3B).

**Table 4:** Multiple regression analysis of the determinants of U‑RBP in the general population, stratified by age groups and adult smoking status.

Participants	*n*	U-RBP (μg/g creatinine)				U-RBP (μg/L)			
		Independent variable	Coefficient (95% CI)	*p*-Value	*r*^2^	Independent variable	Coefficient (95% CI)	*p*-Value	*r*^2^
Children	296	U-Cd	0.217 (0.057, 0.376)	0.007	0.177	U-Cd	0.198 (0.025, 0.375)	0.026	0.300
		Age	–0.100 (–0.127, –0.073)	<0.001		U-Creat	0.756 (0.561, 0.944)	<0.001
						Age	–0.098 (–0.126, –0.069)	<0.001
Adolescents	200	U-Cd	0.313 (0.096, 0.529)	0.005	0.093	U-Cd	0.363 (0.139, 0.587)	0.002	0.409
		BMI	–1.07 (–1.66, –0.48)	<0.001		BMI	–1.07 (–1.65, –0.477)	<0.001
						U-Creat	0.808 (0.549, 1.07)	<0.001
Adults (19–70 years)
All	744	U-Cd	0.125 (0.073, 0.177)	<0.001	0.049	U-Cd	0.116 (0.060, 0.173)	<0.001	0.391
		BMI	–0.546 (–0.786, –0.305)	<0.001		BMI	–0.545 (–0.785, –0.304)	<0.001
						U-Creat	0.846 (0.761, 0.932)	<0.001
Never-smokers	284	U-Cd	0.111 (0.029, 0.193)	0.008	0.034	U-Cd	0.103 (0.007, 0.200)	0.035	0.389
		BMI	–0.577 (–0.982, –0.173)	0.005		BMI	–0.580 (–0.986, –0.174)	0.005
						U-Creat	0.872 (0.733, 1.01)	<0.001
Ever-smokers	460	U-Cd	0.138 (0.068, 0.211)	<0.001	0.052	U-Cd	0.127 (0.052, 0.203)	0.001	0.381
		BMI	–0.515 (–0.978, –0.181)	<0.001		BMI	–0.504 (–0.809, –0.198)
						U-Creat	0.822 (0.709, 0.935)
Adults (> 70 years)
All	98	U-Cd	0.013 (–0.391, 0.188)	0.49	0.005	U-Cd	–0.077 (–0.382, 0.227)	0.6	0.197
						U-Creat	1.21 (0.720, 1.69)	<0.001
Never-smokers	62	U-Cd	–0.076 (–0.406, 0.255)	0.65	0.003	U-Cd	–0.053 (–0.392, 0.286)	0.8	0.259
						U-Creat	1.22 (0.673, 1.76)	<0.001
Ever-smokers	36	U-Cd	–0.128 (–0.699, 0.443)	0.65	0.006	U-Cd	–0.114 (–0.731, 0.503)	0.7	0.092
						U-Creat	1.19 (0.156, 2.21)	0.025
CI, confidence interval.									

## Discussion

We conducted the present study using data from six epidemiological studies that included participants of different ages from the general population living in Brussels and the southern part of Belgium. The U-Cd levels we report here are very similar to those reported in Canada (Health Canada 2010) and in several European countries (Aguilera et al. 2010; Dhooge et al. 2010; Pennemans et al. 2011). However, these U-Cd concentrations were about two or three times higher than those observed in the United States (Centers for Disease Control and Prevention 2009) or Germany (Wilhelm et al. 2005). Our findings show that, at these low levels of exposure, U-Cd increases with age in a nonmonotonic manner. The evolution of U-Cd over the lifetime is characterized by a transient increase during childhood, a decrease during adolescence, and a progressive rise until the age of 60 years, at which time the U-Cd concentration levels off or decreases. These variations were amplified in U-Creat–adjusted values as a result of age-related variations in U-Creat, which peaks at puberty and progressively declines over the rest of life in both men and women. In adults, U-Cd curves were systematically shifted to higher values in comparisons of women and men and comparisons of current and never-smokers. In contrast, U-Cd did not vary significantly with gender in children or adolescents, or with past smoking in adults. In addition, U-Cd was consistently associated with U-RBP in all age groups, except the elderly.

The lifetime patterns of U-Cd variations we observed are in discordance with mathematical models of Amzal et al. (2009) that predicted a monotonic curvilinear increase of U-Cd with age until it levels off around 60–70 years of age. Our findings are also not consistent with data from autopsy studies or from models describing an almost linear increase of K-Cd from birth until 50–60 years of age, at which a plateau or a decrease is seen (Benedetti et al.1999). Interestingly, models that take into account the variations in kidney weight with age have predicted a small decrease of both K-Cd and U-Cd during puberty (Friberg et al. 1974; Ruiz et al. 2010), a phenomenon similar to that observed with U-Cd in our study. Clearly, values of U-Cd observed during childhood or adolescence cannot be interpreted as a reflection of K-Cd. Children at 8 years of age have about the same U-Cd levels as 40-year-old never-smokers. The patterns of U-Cd observed in our study are similar to K-Cd patterns between 20 and 60 years of age. This similarity holds even in quantitative terms because the relative increases of U-Cd for both men and women during that period (3-fold for U-Cd per liter and 4-fold for U-Cd per gram of creatinine) are in the same range as that observed with K-Cd in the nonsmoking general population (Benedetti et al. 1999). However, it is necessary to exercise caution before interpreting this similarity as evidence that U-Cd reflects a concomitant increase of K-Cd. Unexpectedly, the U-Cd increase with age did not differ between former smokers and never-smokers. In addition, the difference in U-Cd between current smokers and never-smokers remained fairly constant over the lifetime, instead of increasing as described for K-Cd (Hahn et al. 1987). These results are in agreement with a previous study in which Ikeda et al. (2005) observed no significant difference in U-Cd between former smokers and never-smokers. Our results suggest that the higher U-Cd observed among current smokers reflects the higher intake of Cd from tobacco smoke rather than the increasing Cd renal burden from chronic smoking.

These discordant patterns of U-Cd and K-Cd with age can probably be explained by the two basic mechanisms that govern the urinary excretion of Cd and whose contributions vary with the level of Cd exposure and the integrity of renal function. The first mechanism, the release or secretion of Cd accumulated in the kidney, is probably responsible for the parallel rise of U-Cd with the Cd renal burden described in industrial workers and experimental animals with a preserved renal function (Nordberg et al. 2007). This mechanism explains why, in workers with no tubular dysfunction, U-Cd remains stable and elevated many years after exposure is discontinued (Friberg 1984). The second mechanism is the glomerular filtration of Cd-MT, which is followed by the excretion of Cd-MT that is not reabsorbed by the proximal tubule. In this mechanism, the urinary output of Cd is determined both by the amount of circulating Cd—thus by the Cd intake—and by the capacity of the kidneys to filter and reabsorb LMW proteins. Our data suggest that this second mechanism is predominant in determining U-Cd when the Cd body burden is very low. In that case, there is a minimal release of Cd from kidneys into urine and also a minimal contribution of the body burden to circulating Cd. This may explain why children have U-Cd levels similar to those of middle-aged adults; their Cd intake is higher than that of adults (European Food Safety Authority 2012). This second mechanism also seems to operate during adulthood, as evidenced by increased U-Cd with increasing U-RBP or U-Alb, presumably a consequence of coexcretion mechanisms (Akerstrom et al. 2012; Chaumont et al. 2012; Haddam et al. 2011).

The only period of life when U-Cd might reflect K-Cd is between puberty and the age of 60–70 years, when U-Cd increases with age in a manner similar to that of as K-Cd, regardless of the physiological variations in creatinine and protein excretion. Even at that stage, the significance of U-Cd on an individual basis remains uncertain. The patterns of U-Cd increasing with age in current smokers and former smokers are not consistent with those described for K-Cd in autopsy studies (Benedetti et al. 1999). These findings, combined with the evidence of a coexcretion mechanism of U-Cd with urinary proteins, suggest that even in adults, U-Cd might be more a reflection of current Cd intake and protein reabsorption capacity of the kidney. Actually, the kidney’s protein reabsorption capacity might lead to an inverse relationship between U-Cd and K-Cd because for a similar intake, less tubular reabsorption of the metal means less accumulation in the kidney. This situation is reminiscent of the decline of the K-Cd in subjects who developed kidney damage as a result of high industrial or environmental exposure to the metal (Nordberg et al. 2007). Likewise, the decrease of U-Cd observed in the elderly might mirror a parallel decline of the Cd renal burden, even though other aging-related explanations are possible, such as a reduced Cd intake or a lower glomerular filtration of Cd-MT.

Our study has several limitations. Because of ethical and practical reasons, it was not possible to collect blood and timed urine samples in all studied groups. Therefore, we could not measure blood Cd, a biomarker of current Cd exposure. A comparison of the variations of U-Cd and blood Cd over a lifetime would have been particularly interesting to determine the relative sensitivities of these two biomarkers to physiological changes occurring during development and aging. In addition, we could not calculate creatinine clearance to estimate the GFR, a factor that positively influences the renal elimination of Cd (Weaver et al. 2011).

## Conclusions

Our study shows that the curvilinear relationship between U-Cd and K-Cd described in industrial workers and assumed in recent models does not hold for the entire general population with a low environmental exposure to Cd. Over a lifetime, U-Cd shows age-related variations that appear to be largely determined by recent Cd intake and by the renal handling of proteins, particularly LMW proteins. These findings are particularly relevant for epidemiological studies of health risks associated with low environmental exposures to Cd. Observations in these studies based on U-Cd would be substantiated by the use of cumulative intake indicators that are unlikely to be confounded by recent Cd exposure and physiological variations in renal elimination of the metal. Various indicators might be used for that purpose based on, for instance, residence time in the studied area, consumption of locally produced foods, or Cd dietary intake estimated from food contamination data.

## Supplemental Material

(1.4 MB) PDF

## References

[r1] Aguilera I, Daponte A, Gil F, Hernandez AF, Godoy P, Pla A (2010). Urinary levels of arsenic and heavy metals in children and adolescents living in the industrialized area of Ria of Huelva (SW Spain).. Environ Int.

[r2] Akerstrom M Sallsten G Lundh T Barregard L 2013 Associations between urinary excretion of cadmium and proteins in a nonsmoking population: renal toxicity or normal physiology? Environ Health Perspect 121 187 191; 10.1289/ehp.1205418. 23128055 PMC3569687

[r3] Amzal B Julin B Vahter M Wolk A Johanson G Åkesson A 2009 Population toxicokinetic modeling of cadmium for health risk assessment. Environ Health Perspect 117 1293 1301; 10.1289/ehp.0800317. 19672411 PMC2721875

[r4] Barr DB Wilder LC Caudill SP Gonzalez AJ Needham LL Pirkle JL 2005 Urinary creatinine concentrations in the U.S. population: implications for urinary biologic monitoring measurements. Environ Health Perspect 113 192 200; 10.1289/ehp.7337. 15687057 PMC1277864

[r5] Benedetti JL, Samuel O, Dewailly E, Gingras S, Lefebvre MA (1999). Levels of cadmium in kidney and liver tissues among a Canadian population (province of Quebec).. J Toxicol Environ Health A.

[r6] Bernard A, Lauwerys R (1983). Continuous-flow system for the automation of latex immunoassay by particle counting.. Clin Chem.

[r7] Bernard A, Nickmilder M, Voisin C (2008). Outdoor swimming pools and the risks of asthma and allergies during adolescence.. Eur Resp J.

[r8] Buchet JP, Lauwerys R, Roels H, Bernard A, Bruaux P, Claeys F (1990). Renal effects of cadmium body burden of the general population.. Lancet.

[r9] Centers for Disease Control and Prevention (2009). Fourth National Report on Human Exposure to Environmental Chemicals. Atlanta, GA:Centers for Disease Control and Prevention.. http://www.cdc.gov/exposurereport/pdf/fourthreport.pdf.

[r10] Chaumont A De Winter F Dumont X Haufroid V Bernard A, 2011 The threshold level of urinary cadmum associated with increased urinary excretion of retinol-binding protein and β_2_-microglobulin: a re-assessment in a large cohort of nickel-cadmium battery workers. Occup Environ Med 68 257 264 20935291 10.1136/oem.2009.054122 PMC3060309

[r11] Chaumont A, Nickmilder M, Dumont X, Lundh T, Skerfving S, Bernard A (2012). Associations between proteins and heavy metals in urine at low environmental exposures: evidence of reverse causality.. Toxicol Lett.

[r12] Dhooge W, Den Hond E, Koppen G, Bruckers L, Nelen V, Van De Mieroop E (2010). Internal exposure to pollutants and body size in Flemish adolescents and adults: associations and dose–response relationships.. Environ Int.

[r13] European Food Safety Authority 2012 Cadmium dietary exposure in the European population. EFSA J 10 2551; 10.2903/j.efsa.2012.2551.

[r14] Fierens S, Mairesse H, Heilier JF, De Burbure C, Focant JF, Eppe G (2003). Dioxin/polychlorinated biphenyl body burden, diabetes and endometriosis: findings in a population-based study in Belgium.. Biomarkers.

[r15] Friberg L (1984). Cadmium and the kidney.. Environ Health Perspect.

[r16] Friberg L, Piscator M, Nordberg GF, Kjellstrom T (1974). Cadmium in the Environment. 2nd ed.

[r17] Haddam N Samira S Dumont X Taleb A Lison D Haufroid V 2011 Confounders in the assessment of the renal effects associated with low-level urinary cadmium: an analysis in industrial workers. Environ Health 10 37; http://www.ehjournal.net/content/10/1/37 21569589 10.1186/1476-069X-10-37 PMC3118317

[r18] Hahn R, Ewers U, Jermann E, Freier I, Brockhaus A, Schlipköter HW (1987). Cadmium in kidney cortex of inhabitants of North-West Germany: its relationship to age, sex, smoking and environmental pollution by cadmium.. Int Arch Occup Environ Health.

[r19] Hare RS (1950). Endogenous creatinine in serum and urine.. Proc Soc Exp Biol Med.

[r20] Harrell FE (2001).

[r21] Health Canada (2010).

[r22] Ikeda M, Moriguchi J, Ezaki T, Fukui Y, Ukai H, Okamoto S (2005). Smoking-induced increase in urinary cadmium levels among Japanese women.. Int Arch Occup Environ Health.

[r23] Järup L, Åkesson A (2009). Current status of cadmium as an environmental health problem.. Toxicol Appl Pharmacol.

[r24] Lauwerys R, Amery A, Bernard A, Bruaux P, Buchet JP, Claeys F (1990). Health effects of environmental exposure to cadmium: objectives, design and organization of the Cadmibel study: a cross-sectional morbidity study carried out in Belgium from 1985 to 1989.. Environ Health Perspect.

[r25] Nilsson U, Schütz A, Bensryd I, Nilsson A, Skerfving S, Mattsson S (2000). Cadmium levels in kidney cortex in Swedish farmers.. Environ Res.

[r26] Nordberg GF, Fowler BA, Nordberg M, Friberg LT (2007). Handbook on the Toxicology of Metals. 3rd ed.

[r27] Orlowski C, Piotrowski JK, Subdys JK, Gross A (1998). Urinary cadmium as indicator of renal cadmium in humans: an autopsy study.. Hum Exp Toxicol.

[r28] Pennemans V De Winter LM Munters E Nawrot TS Van Kerkhove E Rigo JM 2011 The association between urinary kidney injury molecule 1 and urinary cadmium in elderly during long-term, low-dose cadmium exposure: a pilot study. Environ Health 10 77; 10.1186/1476-069X-10-77. 21888673 PMC3176151

[r29] Roels H, Lauwerys R, Dardenne AN (1983). The critical level of cadmium in human renal cortex: a reevaluation.. Toxicol Lett.

[r30] Roels HA, Lauwerys RR, Buchet JP, Bernard A, Chettle DR, Harvey TC (1981). *In viv*o measurement of liver and kidney cadmium in workers exposed to this metal: its significance with respect to cadmium in blood and urine.. Environ Res.

[r31] Ruiz P, Mumtaz M, Osterloh J, Fisher J, Fowler BA (2010). Interpreting NHANES biomonitoring data, cadmium.. Toxicol Lett.

[r32] Voisin C Sardella A, Bernard. 2013. Risks of new-onset allergic sensitization and airway inflammation after early age swimming in chlorinated pools. Int J Hyg Environ Health; 10.1016/j.ijheh.2013.03.004. [Online 21 March 2013] 23601779

[r33] Voisin C, Sardella A, Marcucci F, Bernard A (2010). Infant swimming in chlorinated pools and the risks of bronchiolitis, asthma and allergy.. Eur Resp J.

[r34] Weaver VM, Kim NS, Jaar BG, Schwartz BS, Parsons PJ, Steuerwald AJ (2011). Associations of low-level urine cadmium with kidney function in lead workers.. Occup Environ Med.

[r35] Wilhelm M, Eberwein G, Hölzer J, Begerow J, Sugiri D, Gladtke D (2005). Human biomonitoring of cadmium and lead exposure of child-mother pairs from Germany living in the vicinity of industrial sources (hot spot study NRW).. J Trace Elem Med Biol.

