# Supplementary material for: Associations of Urinary Cadmium with Age and Urinary Proteins: Further Evidence of Physiological Variations Unrelated to Metal Accumulation and Toxicity
Source: Environ Health Perspect. 2013 Jun 7;121(9):1047–53. doi: 10.1289/ehp.1306607 (PMC3764089; doi:10.1289/ehp.1306607)
Supplement: (1.4 MB) PDF [file ehp.1306607.s001.pdf]

Supplemental Material

Associations of Urinary Cadmium with Age and Urinary Proteins: Further Evidence of Physiological Variations Unrelated to Metal Accumulation and Toxicity

Agnes Chaumont, Catherine Voisin, Gladys Deumer, Vincent Haufroid, Isabella Annesi-Maesano, Harry Roels, Lutgarde Thijs, Jan Staessen, and Alfred Bernard

Table of contents

Analytical Performances of Methods for Measuring Urinary Cd **Page 2**

Figure S1: Linear association between U-Cd and age in the two different studies performed among Belgian adult nonsmokers in 1985-1989 and 2001-2002. **Page 3**

Figure S2: Associations between urinary biomarkers levels and age according to the gender in the nonsmoking population. **Page 4**

References **Page 5**

Analytical Performances of Methods for Measuring Urinary Cd

Cd was measured in urine by means of inductively coupled argon plasma mass spectrometry (ICP-MS) with an Agilent 7500 instrument (Agilent Technologies. Santa Clara, California, USA). Using this ISO15189 certified method, the laboratory has obtained successful proficiency results in external quality assessment schemes with certificates awarded by the Institute for Occupational, Environmental and Social Medicine of the University of Erlangen, Germany (G-EQUAS program) for both occupational and environmental levels during the period of urine analyses (2001-2012) and high performances achieved in the PCI and QMEQAS program organized by the Institut National de Santé Publique, Québec, with z'-score regularly comprised between -1.00 and +1.00. Certificates from G-EQUAS program are available from 1997 until 2013. For instance, results obtained with the G-EQUAS 49/2012 certified controls were as follows: for parameter 74A, our result: 0.30 µg/L, target: 0.30 µg/L, tolerance range 0.18-0.42 µg/L and for parameter 74B, our result: 0.78 µg/L, target: 0.81 µg/L, tolerance range 0.60-1.02 µg/L. Results from the QMEQAS 2012-03 round demonstrated that our analyses by ICP-MS were unaffected by the interference from molybdenum (Mo) (Akerstrom et al. 2013, Jarrett et al. 2008). In an urine sample containing about 78 µg/L of Mo (about the 80th percentile of values in the general population of Belgium), the quantification of Cd at a concentration of about 1 µg/L (9.49 nmol/L) was associated with a z'-score of about -0.40, indicating again the absence of overestimation. Similar performances were achieved for previous years for both G-EQUAS and QMEQAS programs. Results of quality control programmes met the external acceptability criteria and also demonstrated the analyrical proficiency of the U-Cd determation (Claeys et al. 1992).

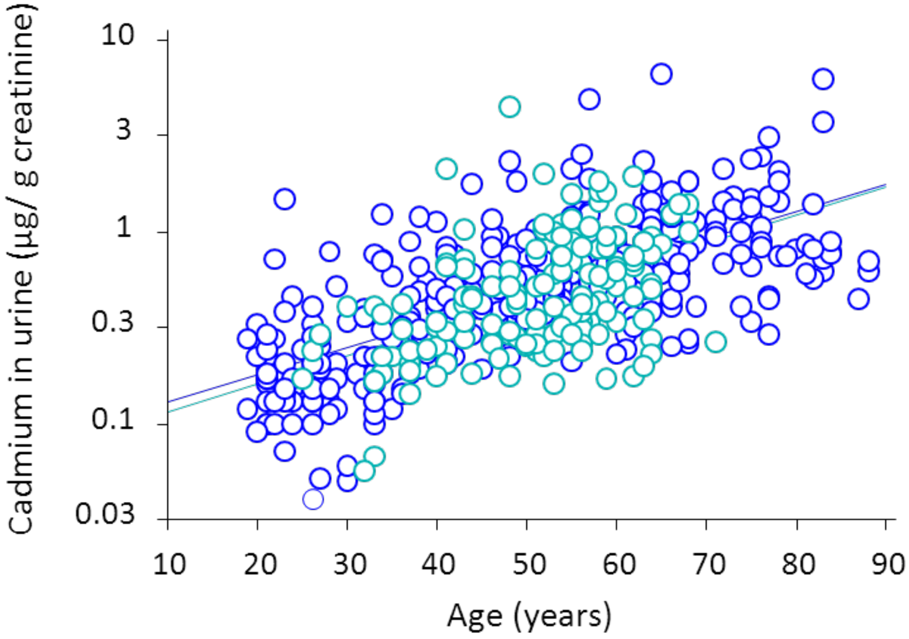

1985-1989 : U-Cd (µg/g creatinine) = -1.01 + 0.014 * Age; R^2 = 0.476

2001-2002 : U-Cd (µg/g creatinine) = -1.09 + 0.015 * Age; R^2 = 0.223

Figure S1: Linear association between U-Cd (µg/g creatinine) and age in the two different studies performed among Belgian adult nonsmokers in 1985-1989 (N=331) and 2001-2002 (N=146).

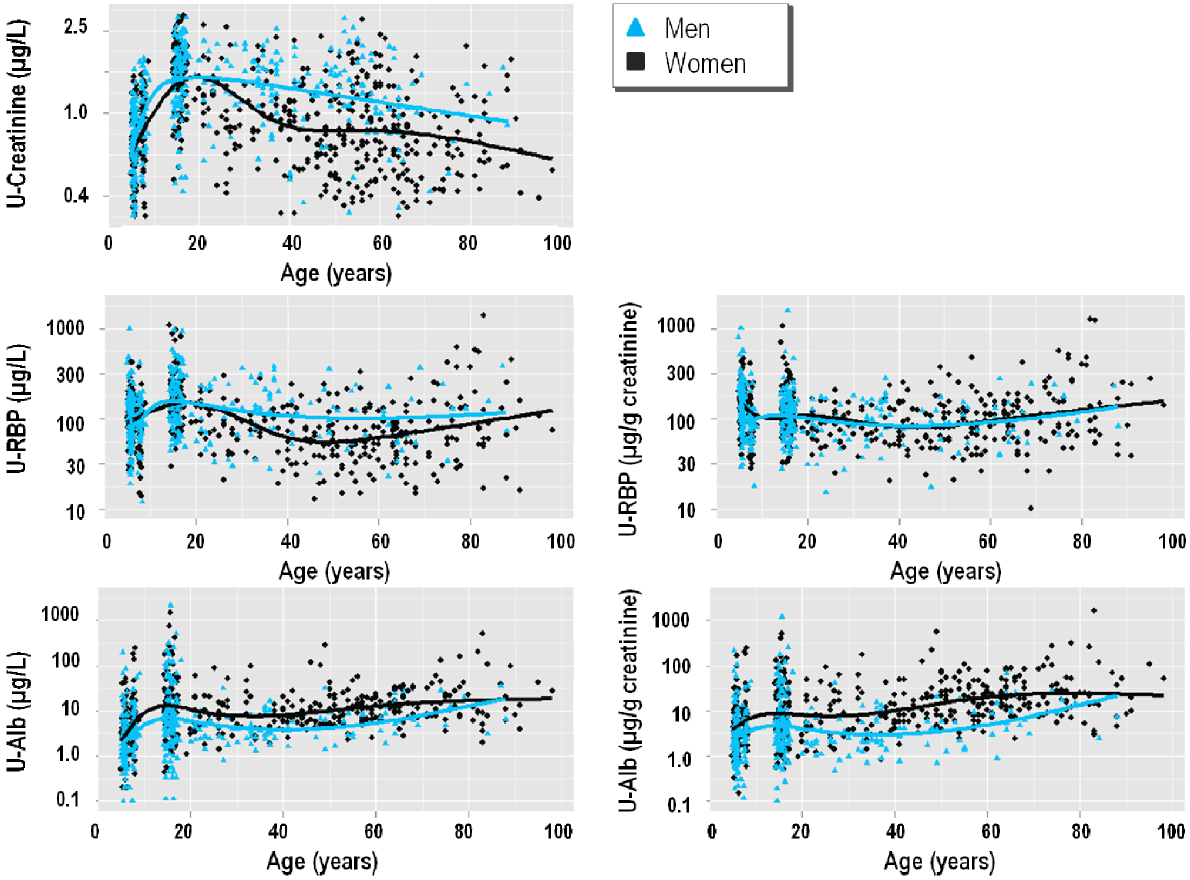

Figure S2

Figure S2: Associations between urinary biomarkers levels and age according to the gender in the nonsmoking population. Data were fitted using natural cubic splines using 4 knots (at percentiles 20th, 40th, 60th and 80th) for U-RBP and 3 knots (at percentiles 25th, 50th and 75h) for U-Alb and U-Creat. The solid lines represent mean U-Cd concentrations in women (light blue, n=494) and men (blue, n=346), respectively.

References

Akerstrom M, Lundh T, Barregard L, Sallsten G. 2013. Effect of molybdenum oxide interference on urinary cadmium analyses. Int Arch Occup Environ Health. DOI 10.1007/s00420-013-0858-9.

Claeys F, Ducoffre G, F. Sartor, and H. Roels. 1992. Analytical quality control of cadmium and lead in blood and cadmium in urine: results of its implementation during a five-year epidemiological study" in Cadmium in the Human Environment: Toxicity and Carcinogenicity, Ed. GF Nordberg, RFM Herber, & L Alessio,Lyon, International Agency for Research on Cancer (IARC) pp. 83-92.

Jarrett JM, Xiao G, Caldwell KL, Henahan D, Shakirova G, Jones RL. 2008. Eliminating molybdenum interference in urine cadmium biomonitoring using ICP-DRC-MS. J Anal At Spectom 23, 962-967.
